# Hydrothermal synthesis of MnO_2_/CNT nanocomposite with a CNT core/porous MnO_2 _sheath hierarchy architecture for supercapacitors

**DOI:** 10.1186/1556-276X-7-33

**Published:** 2012-01-05

**Authors:** Hui Xia, Yu Wang, Jianyi Lin, Li Lu

**Affiliations:** 1 School of Materials Science and Engineering, Nanjing University of Science and Technology, 200 Xiao Ling Wei, Nanjing, 210094, China; 2 Institute of Chemical and Engineering Science (ICES), 1 Pesek Road, Jurong Island, 627833, Singapore; 3 Department of Mechanical Engineering, National University of Singapore, 9 Engineering Drive 1, 117576, Singapore

## Abstract

MnO_2_/carbon nanotube [CNT] nanocomposites with a CNT core/porous MnO_2 _sheath hierarchy architecture are synthesized by a simple hydrothermal treatment. X-ray diffraction and Raman spectroscopy analyses reveal that birnessite-type MnO_2 _is produced through the hydrothermal synthesis. Morphological characterization reveals that three-dimensional hierarchy architecture is built with a highly porous layer consisting of interconnected MnO_2 _nanoflakes uniformly coated on the CNT surface. The nanocomposite with a composition of 72 wt.% (K_0.2_MnO_2_·0.33 H_2_O)/28 wt.% CNT has a large specific surface area of 237.8 m^2^/g. Electrochemical properties of the CNT, the pure MnO_2_, and the MnO_2_/CNT nanocomposite electrodes are investigated by cyclic voltammetry and electrochemical impedance spectroscopy measurements. The MnO_2_/CNT nanocomposite electrode exhibits much larger specific capacitance compared with both the CNT electrode and the pure MnO_2 _electrode and significantly improves rate capability compared to the pure MnO_2 _electrode. The superior supercapacitive performance of the MnO_2_/CNT nancomposite electrode is due to its high specific surface area and unique hierarchy architecture which facilitate fast electron and ion transport.

## Introduction

In recent years, manganese oxides have attracted considerable research interest due to their distinctive physical and chemical properties and wide applications in catalysis, ion exchange, molecular adsorption, biosensor, and energy storage [1-8]. Specifically, manganese dioxide [MnO_2_] has been considered as a promising electrode material for supercapacitors because of its low cost, environmental benignity, and excellent capacitive performance in aqueous electrolytes [9-15]. In aqueous electrolytes, the charging mechanism of MnO_2 _may be described by the following reaction [10]:

(1) $$\text{Mn}{\text{O}}_{2}+{\text{M}}^{+}+{\text{e}}^{-}\Leftrightarrow \text{MnOOM},$$

where M represents protons (H^+^) and/or alkali cations such as K^+^, Na^+^, and Li^+^. The charge storage is based either on the adsorption of cations at the surface of the electrode material or on the intercalation of cations in the bulk of the electrode material. In order to achieve high capacitive performance, a large surface area and a fast ion/electron transport of the electrode material are required. Therefore, extensive research has been focused on the synthesis of nanostructured MnO_2 _as the nanoscale powder, which provides not only a high specific surface area, but also a fast ion and electron transport [16-25]. Various forms of MnO_2 _including one-dimensional (nanorods, nanowires, nanobelts, nanotubes) [16-22], two-dimensional [2-D] (nanosheets, nanoflakes) [23-25], and three-dimensional [3-D] (nanospheres, nanoflowers) [26-28] nanostructures have been synthesized. However, the reported specific capacitance values for the various nanostructured MnO_2 _electrodes are still far below the theoretical value (approximately 1,370 F/g) [29], which may be attributed to the intrinsically poor electronic conductivity of MnO_2_. To improve the capacitive performance of MnO_2_, the key is to add conductive additives to improve the electron transport [30]. Due to their excellent electrical conductivity and high specific surface area, carbon nanotubes [CNTs] are now intensively used with MnO_2 _to make nanocomposites. Recently, MnO_2_/CNT nanocomposites have been prepared by various methods to improve the electrochemical utilization of MnO_2 _and electronic conductivity of the electrode [31-38]. In most studies, once the coated MnO_2 _layer becomes thick, it exhibits a dense structure, which is not beneficial for maximizing the utilization of MnO_2 _as only the surface area is involved in charge storage. However, if the coated MnO_2 _layer is too thin, the specific capacitance of the composite is difficult to be increased as the MnO_2 _loading becomes too low. In previous reports, although the MnO_2 _incorporation improves the capacitance of the CNT assembly, the overall specific capacitance remains typically less than 200 F/g. In order to increase the MnO_2 _loading in the composite while retaining the formation of a nanoscopic MnO_2 _phase, depositing a highly porous MnO_2 _layer on the CNTs could be a strategy to achieve this goal. However, a facile and fast synthesis of a uniformly distributed MnO_2 _porous layer on the CNTs is still a challenge. It could be a beneficial design if one of the nanostructures (nanowire, nanorod, nanoflake, etc.) of MnO_2 _could be transferred onto the CNTs as this hierarchy architecture may be able to provide a large specific surface area (due to the porous feature of the MnO_2 _sheath) and a fast electron and ion transport (due to the support of the CNT core and the formation of the nanoscopic MnO_2 _phase).

In the present work, a facile hydrothermal synthesis has been designed to deposit a uniform and highly porous MnO_2 _layer consisting of interconnected nanoflakes onto the surface of the CNTs. The structure, surface morphology, composition, and specific surface area of the as-prepared nanoflaky MnO_2_/CNT nanocomposites have been fully investigated. The capacitive behaviors of the CNTs, the pure MnO_2_, and the MnO_2_/CNT nanocomposite electrodes were also investigated and compared. The advantages of the present MnO_2_/CNT hierarchy architecture associated with its superior capacitive behaviors were discussed.

### Experimental section

Commercial multiwalled CNTs (20 to 50 nm in diameter, Shenzhen Nanotech Port Co., Ltd., Shenzhen, China) were purified by refluxing the as-received sample in 10 wt.% nitric acid for 12 h. The acid-treated CNTs were then collected by filtration and dried at 120°C for 12 h in vacuum. A typical synthesis process of the MnO_2_/CNT nanocomposite is described as follows. Firstly, 0.1 g CNTs was dispersed in 25 ml deionized [DI] water by ultrasonic vibration for 2 h. Then, 0.3 g KMnO_4 _was added into the above suspension, and the mixed solution was stirred by a magnetic bar for 2 h. After that, the mixed solution was transferred to a 30-mL, Teflon-lined, stainless steel autoclave. The autoclave was sealed and put in an electric oven at 150°C for 6 h and then naturally cooled to room temperature. After the hydrothermal treatment, the resultant samples were collected by filtration and washed with DI water. MnO_2_/CNT nanocomposites were finally dried in an oven at 100°C for 12 h for further characterization. To prepare the MnO_2 _powders, 0.3 g KMnO_4 _and 0.2 mL H_2_SO_4 _(95 wt.%) were placed into 25 mL DI water to form the precursor. The precursor solution was then treated with a hydrothermal reaction in a 30-mL autoclave at 150°C for 4 h.

The crystallographic information of the products was investigated by X-ray diffraction [XRD] (Shimadzu X-ray diffractometer 6000, Cu Kα radiation, Kyoto, Japan) with a scan rate of 2°/min. Morphologies of the acid-treated CNTs, the MnO_2 _powders, and the MnO_2_/CNT nanocomposites were characterized by field emission scanning electron microscopy [FESEM] (Hitachi S4300, Tokyo, Japan). The morphology and structure of the MnO_2_/CNT nanocomposites were further investigated by transmission electron microscopy [TEM] and high-resolution transmission electron microscopy [HRTEM] (JEM-2010, JEOL, Tokyo, Japan). Compositional investigation of the samples was carried out with energy-dispersive X-ray [EDX] spectroscopy (Noran System SIX, Thermo Fisher Scientific, Shanghai, China). The contents of the interlayer water and CNTs of the nanocomposites were determined by thermogravimetric analysis [TGA] (Shimadzu DTG-60H, Kyoto, Japan). Nitrogen adsorption and desorption isotherms at 77.3 K were obtained with a Quantachrome Autosorb-6B (Beijing, China) surface area and a pore size analyzer.

Electrochemical measurements were carried out on three-electrode cells using a Solartron 1287 electrochemical interface combined with a Solartron 1260 frequency response analyzer (Hampshire, United Kingdom). To prepare the working electrode, 80 wt.% of the active material (CNTs, pure MnO_2 _powder, or MnO_2_/CNT nanocomposite), 15 wt.% carbon black, and 5 wt.% polyvinylidene difluoride dissolved in *N*-methylpyrrolidone were mixed to form a slurry. The slurry was pasted onto a Ti foil and dried for 12 h in a vacuum oven. The loading of the working electrode is typically in the range of 2 to 3 mg/cm^2^. A carbon rod was used as the counter electrode, an Ag/AgCl (saturated KCl) electrode was used as the reference electrode, and a 1-M Na_2_SO_4 _solution was used as the electrolyte. Cyclic voltammetry [CV] and electrochemical impedance spectroscopy [EIS] were utilized to evaluate the electrochemical behaviors of the different composite electrodes. CV measurements were carried out between 0 and 0.9 V (vs. Ag/AgCl) at different scan rates ranging from 10 to 100 mV/s. EIS measurements were carried out in a frequency range from 10 kHz to 0.01 Hz with an ac amplitude of 10 mV.

## Results and discussion

**Figure 1:**
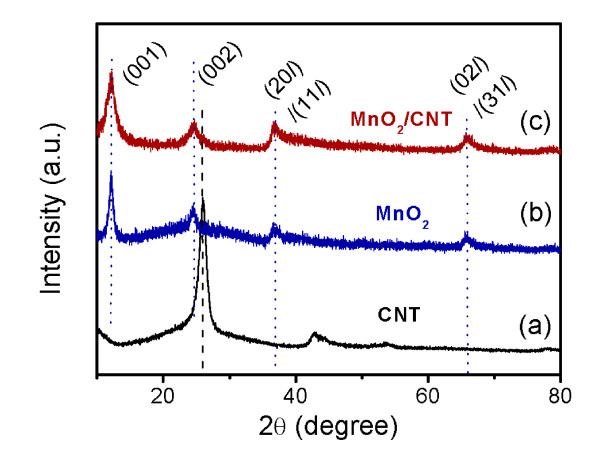
**XRD patterns of the (a) pristine CNTs, (b) pure MnO_2_, and (c) MnO_2_/CNT nanocomposite**.

XRD patterns of the CNTs, the pure MnO_2 _powder, and the MnO_2_/CNT nanocomposite are shown in Figure 1. The XRD pattern of the CNTs shows three diffraction peaks at 26.5°, 43.2° and 54.2° which can be indexed as the (002), (100), and (004) reflections of graphite, respectively [39]. The XRD pattern of the pure MnO_2 _powder synthesized by hydrothermal reaction can be indexed to the monoclinic potassium birnessite (JCPDS number 80-1098), which consists of 2-D, edge-shared MnO_6 _octahedral layers with K^+ ^cations and water molecules in the interlayer space. The two stronger diffraction peaks correspond to (001) and (002) basal reflections, while another two weaker diffraction peaks can be indexed as the (20*l*/11*l*) and (02*l*/31*l*) diffraction bands, respectively [24]. From the XRD pattern of the MnO_2_/CNT nanocomposite, diffraction peaks from the birnessite-type MnO_2 _phase can be observed while the diffraction peaks from the CNTs are not obvious due to the coating of the MnO_2 _layer.

**Figure 2:**
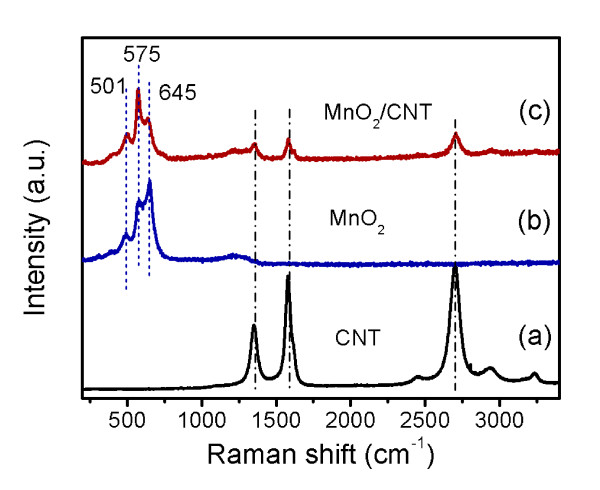
**Raman spectra of the (a) pristine CNTs, (b) pure MnO_2_, and (c) MnO_2_/CNT nanocomposite**.

The structural features of the MnO_2_/CNT nanocomposite were further investigated using Raman measurements as shown in Figure 2. Three Raman bands at 1,577 (G band), 1,327 (D band), and 2,652 cm^-1 ^(2D band) are observed in Figure 2a for the pristine CNTs, which originate from the Raman-active, in-plane atomic displacement E2g mode, disorder-induced features of the CNTs and the overtone of D band [36]. As shown in Figure 2b, three Raman bands located at 501, 575, and 645 cm^-1 ^for the MnO_2 _powder are in good agreement with the three major vibrational features of the birnessite-type MnO_2 _compounds previously reported at 500 to 510, 575 to 585, and 625 to 650 cm^-1 ^[40]. Three Raman bands for the birnessite-type MnO_2 _and three Raman bands for the CNTs can be observed at the same time for the MnO_2_/CNT nanocomposite. Therefore, the results of the Raman measurement agree well with the XRD results confirming that the birnessite-type MnO_2 _has been formed during the hydrothermal treatment with or without the CNTs.

**Figure 3:**
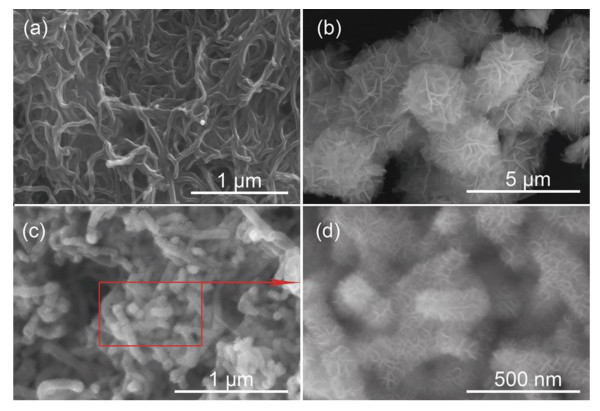
**FESEM images**. FESEM images of **(a) **the pristine CNTs, **(b) **the flower-like MnO_2 _powder synthesized by hydrothermal reaction, and **(c) **the MnO_2_/CNT nanocomposite synthesized by hydrothermal reaction. **(d) **A magnified FESEM image of the MnO_2_/CNT nanocomposite synthesized by hydrothermal reaction.

Morphologies of the CNTs, the birnessite-type MnO_2 _powder, and the MnO_2_/CNT nanocomposite are characterized by FESEM as shown in Figure 3. It can be observed in Figure 3a that the diameter of the CNTs is about 20 to 50 nm. In Figure 3b, it can be seen that the MnO_2 _synthesized by hydrothermal reaction consists of monodisperse microspheres of 2 to 3 μm in diameter. The MnO_2 _microspheres exhibit a flower structure composed of many nanoflakes radiating from the center. Figures 3c,d show the FESEM images of the MnO_2_/CNT nanocomposite at low and high magnifications, respectively. It can be noted that the average diameter of the nanotubes increases for the MnO_2_/CNT nanocomposite compared to the pristine CNTs, indicating that a thin MnO_2 _layer has been coated on the CNT surface. The coated MnO_2 _layer is uniform, exhibiting a highly porous structure.

**Figure 4:**
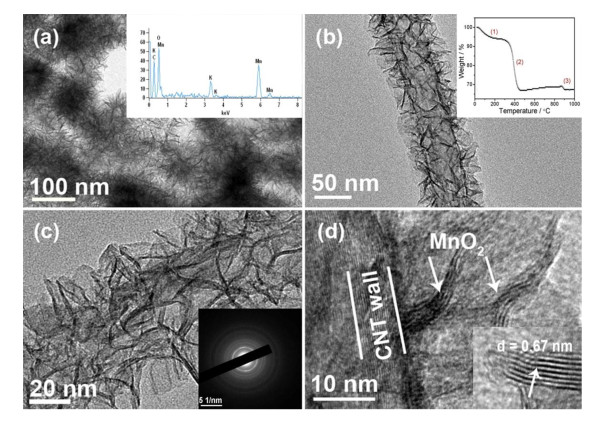
**TEM and HRTEM images**. **(a) **TEM image of the MnO_2_/CNT nanocomposite. **(b) **TEM and **(c) **magnified TEM images of a single CNT coated with a porous MnO_2 _layer. **(d) **HRTEM image of the interface between MnO_2 _and CNT. Inset in (a) shows the EDX spectrum of the MnO_2_/CNT nanocomposite. Inset in (b) shows the TGA spectrum of the MnO_2_/CNT nanocomposite. Inset in (c) shows the ED pattern of the MnO_2_/CNT nanocomposite. Inset in (d) shows the interplanar spacing of MnO_2 _nanoflake grown on the CNT.

TEM images of the MnO_2_/CNT nanocomposite are shown in Figure 4. As shown in Figure 4a, the CNT core and the highly porous MnO_2 _sheath resembling caterpillar-like morphology can be clearly seen. The K/Mn ratio obtained from EDX spectroscopy is about 0.2 as shown in the inset in Figure 4a. Figures 4b,c show the TEM images of a single CNT coated with porous MnO_2 _at different magnifications. It can be seen that the porous MnO_2 _layer is composed of numerous tiny nanoflakes, which are interconnected and uniformly distributed on the surface of the CNT. The interlayer water content and the CNT content can be evaluated from the TGA measurement as shown in the inset in Figure 4b. According to the weight loss of 6% below 250°C, the calculated interlayer water is around 0.33 H_2_O per chemical formula (K_0.2_MnO_2_·0.33H_2_O). The weight loss of 28% at about 400°C is due to the oxidation of CNT in air [36]. Consequently, the composition of the MnO_2_/CNT nanocomposite may be expressed as 72 wt.% (K_0.2_MnO_2_·0.33H_2_O)/28 wt.% CNT. For convenience, MnO_2_/CNT is still used in the following text. The thickness of the porous MnO_2 _layer is estimated to be about 20 nm as shown in Figure 4c. The inset in Figure 4c shows the electron diffraction [ED] pattern of the MnO_2 _nanoflakes on the CNTs. Figure 4d shows the HRTEM image of the interface between the CNT and the MnO_2 _layer. It can be seen that MnO_2 _nanoflakes grow directly from the CNT walls, forming nearly vertically aligned MnO_2 _nanoflake arrays. As shown in the inset in Figure 4e, the interplanar spacing of MnO_2 _nanoflake has been measured to be 0.67 nm, which is in good agreement with approximately 0.7 nm as reported in the literature for birnessite-type MnO_2 _[23,24]. Compared to the self-assembled MnO_2 _nanoflakes of pure MnO_2 _microspheres, these MnO_2 _nanoflakes grown on CNTs are much smaller in dimension, typically with a thickness of less than 5 nm.

The formation mechanism of the present nanoarchitecture is discussed below. When the mixed solution with the CNT suspension and KMnO_4 _is stirred at room temperature, a slow redox reaction between CNTs and KMnO_4 _could take place and can be expressed as:

(2) $$4{\text{MnO}}_{4}^{-}+3\text{C}+{\text{H}}_{2}\text{O}\to 4\text{Mn}{\text{O}}_{2}+{\text{CO}}_{3}^{2-}+2{\text{HCO}}_{3}^{-}.$$

The slow redox reaction usually leads to the precipitation of MnO_2 _nanocrystallines on the surface of the CNTs. When the mixed solution is further undergone through the hydrothermal reaction, the redox reaction continues, but it may not be the major contribution to the later growth of MnO_2 _nanoflakes on the CNTs. In the present experiments, stoichiometric amounts of KMnO_4 _and CNTs were mixed in a solution based on Equation 2 for a hydrothermal reaction. After the hydrothermal reaction, no noticeable decrease in CNTs can be observed from the product, indicating that another reaction for the formation of MnO_2 _may be dominant in the hydrothermal process. Porous MnO_2 _films composed of nanoflakes have been reported to be easily produced in a hydrothermal reaction of KMnO_4 _solution without CNTs [24,25]. The formation of MnO_2 _in such hydrothermal reaction is based on the decomposition of KMnO_4_, which can be expressed as:

(3) $$4{\text{MnO}}_{4}^{-}+2{\text{H}}_{2}\text{O}\to 4\text{Mn}{\text{O}}_{2}+4\text{O}{\text{H}}^{-}+3{\text{O}}_{2}$$

It is speculated that in the present solution system, the decomposition of KMnO_4 _is much faster than the redox reaction between KMnO_4 _and CNTs. During the hydrothermal reaction, the preformed MnO_2 _nanocrystallines may serve as nucleation sites, where the newly formed MnO_2 _nucleus due to the KMnO_4 _decomposition could get deposited on. The flaky morphology is formed due to preferred growth along the ab plane of the layered birnessite-type MnO_2 _[23,24]. Consequently, the CNT core/porous MnO_2 _sheath hierarchy architecture could be easily produced using this simple hydrothermal method.

**Figure 5:**
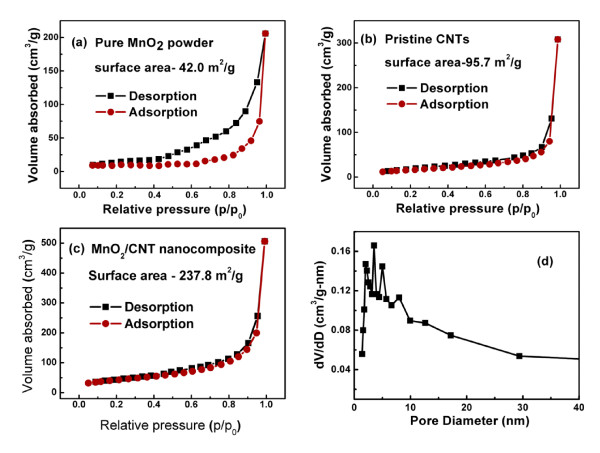
**Nitrogen adsorption-desorption isotherms and BJH pore-size distributions**. Nitrogen adsorption-desorption isotherms of **(a) **the pristine CNTs, **(b) **the MnO_2 _powder, and **(c) **the MnO_2_/CNT nanocomposite. **(d) **BJH pore-size distributions of the MnO_2_/CNT nanocomposite.

The specific surface area and pore size distribution of the MnO_2_/CNT nanocomposite were obtained from an analysis of the desorption branch of N_2 _gas isotherms using the density function theory. As shown in Figure 5c, an isotherm is typical for a mesoporous material with a hysteresis loop at high partial pressures. According to Brunauer-Emmett-Teller [BET] analysis, a total specific surface area of 237.8 m^2^/g is obtained for the MnO_2_/CNT nanocomposite, which is much larger than that of the pure MnO_2 _(42.1 m^2^/g, see Figure 5a) and that of the pristine CNTs (95.7 m^2^/g, see Figure 5b). The Barrett-Joyner-Halenda [BJH] pore size distribution (Figure 5d) indicates that the MnO_2_/CNT nanocomposites exhibit developed mesopores ranging from 2 to 8 nm, which may mainly be attributed to the numerous gaps between the MnO_2 _nanoflakes.

**Figure 6:**
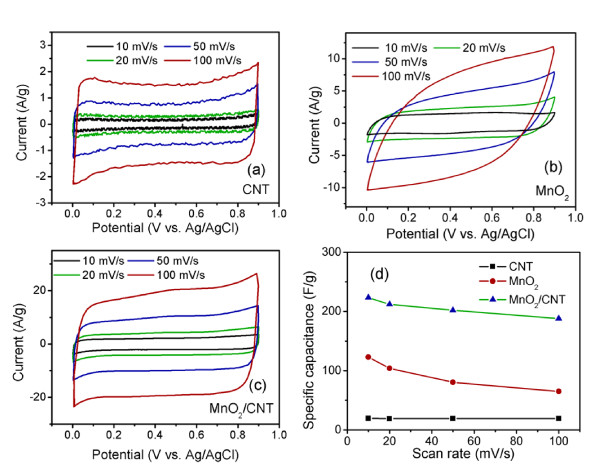
**Cyclic voltammograms and specific capacitance vs. scan rate of the different electrodes**. Cyclic voltammograms for the **(a) **CNT, **(b) **pure MnO_2_, and **(c) **MnO_2_/CNT nanocomposite electrodes in a 1-M Na_2_SO_4 _solution at different scan rates from 10 to 100 mV/s. **(d) **Specific capacitance vs. scan rate of the different electrodes.

The hierarchy architecture and high specific surface area of the MnO_2_/CNT nanocomposite make it promising for applications in catalysis and in energy storage. In the present study, the electrochemical performance of the MnO_2_/CNT nanocomposite as an electrode material in supercapacitors was investigated. Capacitive behaviors of the pristine CNT, the pure MnO_2_, and the MnO_2_/CNT nanocomposite electrodes in a 1-M Na_2_SO_4 _electrolyte at different scan rates are shown in Figure 6. The CV curves of the CNT electrode at different scan rates from 10 to 100 mV/s as shown in Figure 6a exhibit a rectangular shape without obvious redox peaks, indicating an ideal capacitive behavior. However, the specific capacitance of the pure CNT electrode is less than 25 F/g. Figure 6b shows the CV curves of the pure MnO_2 _electrode at different scan rates. The current densities of the CV curves for the pure MnO_2 _electrode increase significantly compared to those for the pure CNT electrode, which indicates that the MnO_2 _electrode can deliver much higher capacitance. However, the rectangularity of the CV curves is significantly distorted as the scan rate increases, especially at a high scan rate of 100 mV/s. The specific capacitance of the MnO_2 _electrode is about 123 F/g at a scan rate of 10 mV/s, while it decreases to 68 F/g at a scan rate of 100 mV/s. Figure 6c shows the CV curves of the MnO_2_/CNT nanocomposite electrode at different scan rates. The current densities of the CV curves for the MnO_2_/CNT nanocomposite electrode are even larger than those for the pure MnO_2 _electrode, indicating higher specific capacitance and higher utilization of MnO_2 _in the MnO_2_/CNT nanocomposite electrode. The specific capacitance of the MnO_2_/CNT nanocomposite electrode is about 223 F/g at a scan rate of 10 mV/s, corresponding to a high specific capacitance of 310 F/g for MnO_2 _alone. CV curves of the MnO_2_/CNT electrode maintain the rectangular shape even at a high scan rate of 100 mV/s with a high specific capacitance of 188 F/g. This is a significantly improved rate capability compared to that for the pure MnO_2 _electrode. Figure 6d compares the specific capacitances at different scan rates for the three types of electrode materials. Although the CNT electrode has a very good rate capability, its specific capacitance is very low due to its surface adsorption charge storage mechanism for double layer capacitors. The pure MnO_2 _electrode exhibits much larger specific capacitance compared with the CNT electrode due to the pseudocapacitance based on faradic redox reactions. However, the rate capability of the pure MnO_2 _electrode is very poor, probably due to its intrinsically poor electronic conductivity and low specific surface area. By combining MnO_2 _and CNT, the MnO_2_/CNT nanocomposite exhibits the two advantages of the two electrode materials, namely a good rate capability and high specific capacitance. Several research groups have also reported the supercapacitive performance of the MnO_2_/CNT nanocomposite [35-38]. Jin et al. [35] reported a MnO_2_/CNT nanocomposite electrode with 65 wt.% MnO_2 _delivering a specific capacitance of 144 F/g at a scan rate of 20 mV/s. The MnO_2_/CNT nanocomposite electrode prepared by Xie et al. [36] was able to deliver a specific capacitance of 205 F/g at a scan rate of 2 mV/s, but only 43.2 F/g at a scan rate of 50 mV/s in a Na_2_SO_4 _electrolyte. The MnO_2_/CNT nanocomposite electrode with 15 wt.% MnO_2 _reported by Yan et al. [38] delivered a specific capacitance of 944 F/g at a scan rate of 1 mV/s based on the mass of MnO_2 _alone or 141 F/g based on the total mass of the composite. From works reported in the literature so far, it appears difficult to achieve a specific capacitance above 200 F/g for the MnO_2_/CNT composite in a Na_2_SO_4 _electrolyte. A high utilization of MnO_2 _can only be achieved with a low mass ratio of MnO_2 _in the composite, which, however, leads to a low specific capacitance of the composite. By increasing the mass ratio of MnO_2 _in the composite with a thicker MnO_2 _layer, the utilization of MnO_2 _is reduced as only the surface area can be used for charge storage. The MnO_2_/CNT nanocomposite electrode in the present study exhibits a superior supercapacitive performance with improved specific capacitance and rate capability compared to MnO_2_/CNT nanocomposites in previous studies. The major difference between the MnO_2_/CNT nanocomposite in the present study and those in previous works is the nanostructure of the MnO_2 _layer. A highly porous MnO_2 _layer composed of interconnected nanoflakes is introduced in the present study instead of a dense MnO_2 _layer composed of closely packed nanocrystallines in previous works. The superior capacitive behavior of the present MnO_2_/CNT nanocomposite electrode may be explained by its unique nanoarchitecture. Firstly, each MnO_2 _nanoflake grows directly on the CNT surface. The CNTs construct a 3-D highly conductive current collector which significantly increases the electronic conductivity of the nanocomposite. Secondly, the large specific surface area and the nanoscopic MnO_2 _phase of the MnO_2_/CNT nanocomposite minimize the solid-state transport distances for both ions and electrons into MnO_2_. This ensures a high utilization of the electrode materials, a high specific capacitance, and a good rate capability. Thirdly, the highly porous structure of the MnO_2 _layer is able to minimize the diffusion distance of the electrolyte to the interior surfaces of MnO_2_, which facilitates better penetration of the electrolyte into the electrode material and enhances the ionic conductivity of the electrode material. With this porous nanostructure of the MnO_2 _layer, the utilization of MnO_2 _can still be high even when the layer becomes thicker. This unique architecture enables the MnO_2_/CNT nanocomposite electrode to have not only a large specific surface, but also a fast electron and ion transport, thus presenting the best electrochemical capacitive performance.

**Figure 7:**
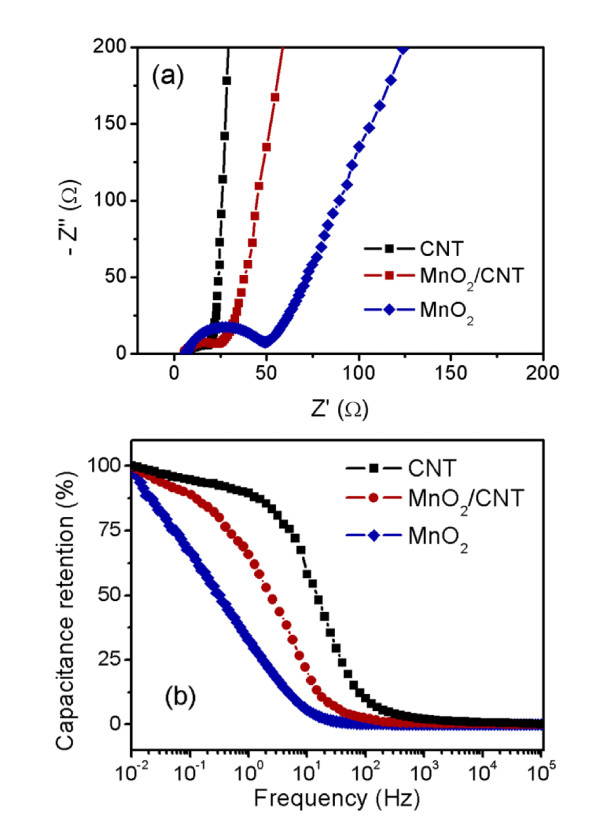
**Nyquist plots and frequency dependence of capacitance retention of the electrodoes**. **(a) **Nyquist plots and **(b) **frequency dependence of capacitance retention of the CNT, pure MnO_2_, and MnO_2_/CNT nanocomposite electrodes.

EIS measurements on the CNT, the pure MnO_2_, and the MnO_2_/CNT nanocomposite electrodes were performed at 0 V vs. Ag/AgCl, and the resulting Nyquist plots are displayed in Figure 7a. The Nyquist plots consist of (1) a high-frequency intercept on the real *Z' *axis, (2) a semicircle in the high-to-medium-frequency region, and (3) a straight line at the very low-frequency region. The high-frequency intercepts for all the three electrodes are almost the same, indicating that the three electrodes have the same combination resistance of ionic resistance of the electrolyte, intrinsic resistance of the active materials, and contact resistance between the active material and the current collector [41]. The semicircle in the high-to-medium-frequency region corresponds to a parallel combination of charge-transfer resistance (*R*_ct_) and double-layer capacitance [42]. It can be seen that the *R*_ct_, which is equal to the diameter of the semicircle, for the three electrodes is in the order of CNT < MnO_2_/CNT < MnO_2_. The *R*_ct _of the MnO_2_/CNT nanocomposite electrode is slightly larger than that of the CNT electrode, but much smaller than that of the pure MnO_2 _electrode. It is speculated that the low *R*_ct _of the MnO_2_/CNT nanocomposite electrode is due to its high specific surface area, which facilitates a faster cation insertion/extraction process into/from the MnO_2 _lattice. For a simple electrode-electrolyte system, the low-frequency straight line should exhibit a slope of 45° if the process is under diffusion control, or a slope of 90° if the system is purely capacitive in nature [43]. The almost vertical line for the CNT electrode here demonstrates a good capacitive behavior without diffusion limitation. The finite slope of the straight line represents the diffusive resistance of electrolyte in the electrode pores and cation diffusion in the host materials [41]. It can be seen that the slope of the straight line for the MnO_2_/CNT nanocomposite electrode is similar to that of the CNT electrode, but much larger than that of the pure MnO_2 _electrode. This observation suggests that the MnO_2_/CNT nancomposite electrode has much lower diffusive resistance compared with the pure MnO_2 _electrode. It is believed that the highly porous MnO_2 _layer decorated on the surface of CNT is able to facilitate the penetration of the electrolyte, leading to fast diffusion of the electrolyte into the pores of the MnO_2 _layer. For the pure MnO_2 _electrode, although the microspheres of the self-assembled MnO_2 _nanoflakes exhibit an open structure at the surface, the center area is quite dense. The latter buffers the electrolyte being diffused into the center area of the sphere. In addition, the dimension of the MnO_2 _nanoflakes for the pure MnO_2 _electrode is much larger compared with that of the MnO_2_/CNT nanocomposite electrode so that the increased diffusion distances for both electrons and ions would also increase the diffusive resistance.

Figure 7b shows the capacitance retention as a function of frequency obtained by taking the real part of the complex capacitance C*(*f*) = 1/[*i*2*πf*Z*(*f*)],where *i*, *f*, and Z* are the imaginary unit, ac frequency, and complex impedance at a frequency, respectively [30,41,44]. For the porous electrode, the frequency response of capacitance may be understood using the parameter 'penetration depth,' *l*' = 1/(*fR*' *C*')^1/2^, where *R*' and *C*' represent the pore resistance and pore capacitance per unit pore length, respectively [44]. At low frequency, when the electrolyte penetration depth is larger than the pore length of the porous electrode, most of the pore surface is utilized, resulting in a maximum capacitance. On the contrary, at high frequency, when the penetration depth is smaller than the pore length, only limited electrode surface is utilized, resulting in a decreased capacitance. As shown in Figure 7b, the capacitance retention for all three electrodes reaches the maximum at very low frequency, starts to decrease as the frequency increases, and finally, goes down to zero at very high frequency. The CNT electrode exhibits an excellent rate capability with capacitance retention of 90% at a frequency of 1 Hz. The pure MnO_2 _electrode however exhibits a poor rate capability with capacitance retention of only 32% at 1 Hz. It can be seen that a significantly improved rate capability can be obtained by combining the MnO_2 _nanoporous sheath with the CNT core. The MnO_2_/CNT nanocomposite is able to retain 65% of its full capacitance at 1 Hz. The significantly improved rate capability of the MnO_2_/CNT nanocomposite electrode could be due to its small charge-transfer resistance and small diffusive resistance, indicating that the unique nanoarchitecture of CNT core/porous MnO_2 _sheath is able to provide fast transport for both ions and electrons.

## Conclusions

MnO_2_/CNT nanocomposites with a unique nanoarchitecture consisting of a CNT core/porous MnO_2 _sheath have been successfully synthesized using a simple hydrothermal treatment. The nanoporous MnO_2 _sheath is composed of interconnected MnO_2 _nanoflakes directly grown from the surface of the CNTs. The birnessite-type MnO_2 _synthesized by the hydrothermal reaction contains 0.2 K^+ ^and 0.3 H_2_O per formula. The nanoflaky MnO_2_/CNT nanocomposite containing 72 wt.% MnO_2 _exhibits a high specific surface area of 237 m^2^/g with a pore distribution of 2 to 8 nm. The MnO_2_/CNT nanocomposite electrode exhibits much higher specific capacitance compared with those of the CNT and the pure MnO_2 _electrodes and a significantly improved rate capability compared to that of the pure MnO_2 _electrode. The high specific capacitance of the MnO_2_/CNT nanocomposite electrode may be attributed to the highly porous structure of the MnO_2 _layer and its high specific surface area, resulting in high utilization of MnO_2_. The significantly improved rate capability of the MnO_2_/CNT nanocomposite electrode compared to that of the pure MnO_2 _electrode could be explained by its small charge-transfer resistance and diffusive resistance obtained from EIS measurements, resulting from its unique hierarchy architecture where the 3-D electron path network constructed by the CNT cores and the nanoporous sheath composed of tiny MnO_2 _nanoflakes facilitate faster electron and ion transport.

## Competing interests

The authors declare that they have no competing interests.

## Authors' contributions

HX synthesized the MnO_2_/CNT nanocomposite and performed the structural and electrochemical characterizations. YW and JYL carried out the BET experiments. LL conceived the study and revised the manuscript. All authors read and approved the final manuscript.
